# Expression profiling of skeletal muscle following acute and chronic β_2_-adrenergic stimulation: implications for hypertrophy, metabolism and circadian rhythm

**DOI:** 10.1186/1471-2164-10-448

**Published:** 2009-09-23

**Authors:** Michael A Pearen, James G Ryall, Gordon S Lynch, George EO Muscat

**Affiliations:** 1 Institute for Molecular Bioscience, The University of Queensland, Queensland 4072, Australia; 2 Basic and Clinical Myology Laboratory, Department of Physiology, The University of Melbourne, Victoria 3010, Australia

## Abstract

**Background:**

Systemic administration of β-adrenoceptor (β-AR) agonists has been found to induce skeletal muscle hypertrophy and significant metabolic changes. In the context of energy homeostasis, the importance of β-AR signaling has been highlighted by the inability of β_1-3_-AR-deficient mice to regulate energy expenditure and susceptibility to diet induced obesity. However, the molecular pathways and gene expression changes that initiate and maintain these phenotypic modulations are poorly understood. Therefore, the aim of this study was to identify differential changes in gene expression in murine skeletal muscle associated with systemic (acute and chronic) administration of the β_2_-AR agonist formoterol.

**Results:**

Skeletal muscle gene expression (from murine tibialis anterior) was profiled at both 1 and 4 hours following systemic administration of the β_2_-AR agonist formoterol, using Illumina 46K mouse BeadArrays. Illumina expression profiling revealed significant expression changes in genes associated with skeletal muscle hypertrophy, myoblast differentiation, metabolism, circadian rhythm, transcription, histones, and oxidative stress. Differentially expressed genes relevant to the regulation of muscle mass and metabolism (in the context of the hypertrophic phenotype) were further validated by quantitative RT-PCR to examine gene expression in response to both acute (1-24 h) and chronic administration (1-28 days) of formoterol at multiple timepoints. In terms of skeletal muscle hypertrophy, attenuation of myostatin signaling (including differential expression of myostatin, activin receptor IIB, phospho-Smad3 etc) was observed following acute and chronic administration of formoterol. Acute (but not chronic) administration of formoterol also significantly induced the expression of genes involved in oxidative metabolism, including hexokinase 2, sorbin and SH3 domain containing 1, and uncoupling protein 3. Interestingly, formoterol administration also appeared to influence some genes associated with the peripheral regulation of circadian rhythm (including nuclear factor interleukin 3 regulated, D site albumin promoter binding protein, and cryptochrome 2).

**Conclusion:**

This is the first study to utilize gene expression profiling to examine global gene expression in response to acute β_2_-AR agonist treatment of skeletal muscle. In summary, systemic administration of a β_2_-AR agonist had a profound effect on global gene expression in skeletal muscle. In terms of hypertrophy, β_2_-AR agonist treatment altered the expression of several genes associated with myostatin signaling, a previously unreported effect of β-AR signaling in skeletal muscle. This study also demonstrates a β_2_-AR agonist regulation of circadian rhythm genes, indicating crosstalk between β-AR signaling and circadian cycling in skeletal muscle. Gene expression alterations discovered in this study provides insight into many of the underlying changes in gene expression that mediate β-AR induced skeletal muscle hypertrophy and altered metabolism.

## Background

Previous studies have demonstrated that chronic administration of β-adrenoceptor (β-AR) agonists (particularly β_2_-AR agonists) can increase myofibrillar protein content and thus induce skeletal muscle hypertrophy in mammals [1-3]. This β_2_-AR induced hypertrophy is believed to be a result of decreased proteolysis coupled with increased protein synthesis [4-9]. The ubiquitin-proteasome signaling [8,10,11], Ca^2+^-dependent proteolysis [12] and/or calpain-mediated proteolysis [6,13,14] have all been proposed to play a role, however the molecular and cellular pathways altered following β-AR agonist administration remain poorly understood [15].

In addition to hypertrophy, acute exposure of skeletal muscle (and cells) to β-AR agonists has been found to modulate oxidative metabolism, energy expenditure, lipolysis [16-21], glucose transport [22], and glucose oxidation [20]. Skeletal muscle accounts for a large proportion of the body's energy demand and thus plays a pivotal role in insulin sensitivity, blood lipid profile and energy balance [23,24].

Underscoring the importance of β-AR signaling in regulating metabolism, transgenic mice lacking all three β-ARs are susceptible to diet-induced obesity. These animals lack any diet- and cold-induced thermogenic response, indicating that β-ARs play a major role in energy expenditure [25]. Similar to the molecular mechanisms underlying skeletal muscle hypertrophy, our understanding of the pathways regulating the metabolic response to β-AR stimulation have yet to be fully elucidated.

In the context of β-AR signaling and skeletal muscle, we (and others ([26]) have previously demonstrated that acute β-AR signaling markedly and transiently increased the expression of the NR4A subgroup of orphan nuclear receptors (*Nur77, Nurr1 *and *Nor-1*) in skeletal muscle tissue and cultures [27,28]. The induction of the NR4A subgroup was associated with the modulation of critical metabolic genes and cellular metabolism [28,29] ([26-29]. Interestingly, in terms of hypertrophy, knockdown of *Nor-1 *expression, *in vitro*, resulted in a >65 fold increase in the expression of myostatin, a key negative regulator of muscle hypertrophy. Such studies suggest that the NR4A subgroup may mediate some effects of β-AR signaling in skeletal muscle.

To examine skeletal muscle gene expression following acute β-AR stimulation, we examined global gene expression in mice using Illumina BeadArrays. Spurlock et al. [30] previously examined global gene expression in skeletal muscle 1 and 10 days after administration of the β_2_-AR agonist clenbuterol. Spurlock et al. identified genes associated with skeletal muscle growth/hypertrophy, including multiple genes associated with proliferation, differentiation, and the recruitment of satellite cells into muscle fibers. Furthermore, they found an increase in the expression of transcriptional and translational initiators responsible for increasing protein synthesis.

We also performed a comprehensive expression analysis of both the acute (1-24 hours) and chronic (1-28 days) effects of β_2_-AR agonist administration. We identified changes in the expression of mRNAs encoding genes associated with skeletal muscle hypertrophy, myoblast differentiation, metabolism, circadian rhythm, transcription, histones, and oxidative stress that occur within 4 hours and alter signaling pathways responsible for the long-term phenotypic footprint of b2-AR activation.

## Results

### Acute systemic administration of the β_2_-adreneroreceptor agonist, formoterol, induces widespread changes in gene expression in skeletal muscle

The entire data set is available via Gene Expression Omnibus (accession number GSE15793). Expression profiling was performed on 16 mice in total using 46K Illumina Sentrix BeadArray chips. Skeletal muscle samples from 16 independent animals were removed at 1 and 4 hours following a single *i.p. *injection of the β_2_-AR agonist, formoterol, or saline (vehicle control). Each timepoint consisted of eight animals with four saline and four formoterol treated animals. The tibialis anterior muscle was chosen for all analyses as it contains predominantly type II fibers, and (in rodents) is known to exhibit marked increases in protein content and lean mass (hypertrophy) in response to β-AR agonist administration [31-35].

Using a p value cutoff of p < 0.05 (see methods for full statistical analysis) and a fold change cutoff of 1.85, at one hour following formoterol administration, 23 probes were significantly altered and 112 probes were significantly altered at four hours. Significant annotated genes from both timepoints are shown in table 1. Significant non-annotated genes (Riken cDNAs and hypothetical proteins) are included in Table 2.

**Table 1:** Significant differentially expressed genes in skeletal muscle after acute systemic administration of β_2_-AR agonist

**Gene Name**	**Genebank ID**	**1 HOUR**		**4 HOURS**		**Potential Relevant Function**
				
		**change**	**p-value**	**change**	**p-value**	
**Growth and myoblast differentiation related**						
Integrin beta 1 binding protein 3 (Itgb1bp3)	XM_125745.1	**+ 3.28**	**NS**	**+ 3.84**	**0.023**	Regulation of terminal myogenesis
Hairy and enhancer of split 1 (Drosophila) (Hes1)	NM_008235.2	**+ 3.58**	**0.021**	**+ 1.02**	**NS**	Possible negative regulator of myogenesis
Small mothers against decapentaplegic homolog 3 (Smad3)	NM_016769	**+ 1.49**	**NS**	**+ 2.20**	**0.006**	Myoblast differentiation
TG interacting factor (Tgif)	NM_009372.2	**+ 1.53**	**NS**	**+ 2.04**	**0.033**	Smad corepressor
Inhibitor of DNA binding 1 (Idb1)	NM_010495.1	**+ 1.79**	**NS**	**+ 1.92**	**0.024**	Myoblast differentiation
Fibroblast growth factor 1 (Fgf1)	NM_010197.2	**+ 1.27**	**NS**	**+ 1.92**	**0.006**	Myoblast differentiation
Signal transducer and activator of transcription 3 (Stat3)	NM_011486.2	**+ 1.10**	**NS**	**+ 1.89**	**0.028**	Myocyte hypertrophy
Small mothers against decapentaplegic homolog 1 (Smad1)	NM_008539.3	**+ 1.08**	**NS**	**- 1.85**	**0.006**	Myoblast differentiation
						
**Metabolism and mitochondrial related**						
PPARγ coactivator 1 alpha (Pgc-1α)	NM_008904.1	**+ 1.53**	**NS**	**+ 5.25**	**0.022**	Mitochondrial biogenesis
Pyruvate dehydrogenase kinase 4 (Pdk4)	NM_013743.1	**+ 1.35**	**NS**	**+ 2.88**	**0.016**	Pyruvate metabolism
Protein phosphatase 1 regulatory subunit 3C (Ppp1r3c)	NM_016854.1	**+ 1.34**	**NS**	**+ 2.55**	**0.021**	Glycogen maintenance
Uncoupling protein 3 (Ucp3)	NM_009464.2	**+ 1.42**	**NS**	**+ 2.37**	**0.017**	Mitochondrial uncoupling
Forkhead box O1 (FoxO1)	NM_019739.2	**- 1.03**	**NS**	**+ 2.29**	**0.025**	Lipid metabolism
Kinesin family member 1B (Kif1b), transcript variant 1	NM_008441.1	**1.00**	**NS**	**- 2.28**	**0.034**	Mitochondrial transport
Hexokinase 2 (Hk2)	NM_013820.1	**+ 1.53**	**NS**	**+ 2.18**	**0.006**	Glycolysis
Phosphomevalonate kinase (Pmvk)	NM_026784.1	**+ 1.31**	**NS**	**+ 2.07**	**0.012**	Cholesterol/steroid synthesis
Plasma membrane associated protein (S3-12)	NM_020568.1	**- 1.02**	**NS**	**+ 2.03**	**0.041**	Lipid droplet protein
Lipin 1 (Lpin1)	NM_015763	**+ 1.16**	**NS**	**+ 2.03**	**0.011**	Lipid metabolism
ATPase, H+ transporting, V1 subunit B, isoform 2 (Atp6v1b2)	NM_007509.2	**+ 1.19**	**NS**	**+ 2.02**	**0.012**	ATPase
Sorbin and SH3-domains containing 1 (Sorbs1)	NM_009166	**+ 1.38**	**NS**	**+ 2.01**	**0.006**	Insulin signaling
Scavenger receptor class B member 1 (Scarb1)	NM_016741.1	**- 1.40**	**NS**	**- 1.91**	**0.008**	Regulation of blood lipids
Peroxisome proliferator activator receptor delta (Pparδ)	NM_011145	**+ 1.06**	**NS**	**+ 1.87**	**0.040**	Lipid metabolism
						
**Regulation of circadian rhythm**						
Nuclear factor, interleukin 3, regulated (Nfil3)	NM_017373.2	**+ 2.06**	**0.020**	**+ 5.32**	**0.014**	Negative regulation of circadian clock
D site albumin promoter binding protein (Dbp)	NM_016974.1	**- 4.59**	**0.015**	**- 3.60**	**0.021**	Regulation of circadian rhythms
Cryptochrome 2 (Cry2)	NM_009963.3	**+ 1.25**	**NS**	**+ 2.78**	**0.005**	Regulation of circadian rhythms
						
**Transcriptional activation**						
FBJ osteosarcoma oncogene (Fos)	NM_010234.2	**+ 5.17**	**0.041**	**+ 1.40**	**NS**	Early stress response
Kruppel-like factor 4 (gut) (Klf4)	NM_010637.1	**+ 3.97**	**0.015**	**+ 2.21**	**0.039**	Anti-proliferative
cAMP responsive element modulator (Crem)	NM_013498.1	**+ 1.65**	**0.025**	**+ 3.50**	**0.004**	Complex transcriptional regulation
CCAAT/enhancer binding protein beta (Cebpb)	NM_009883.1	**+ 1.70**	**NS**	**+ 3.41**	**0.007**	Negative regulator of cardiac hypertrophy
Nuclear receptor subfamily 4, group A, member 2 (Nurr1)	NM_013613.1	**+ 3.21**	**0.015**	**+ 1.91**	**0.044**	Complex transcriptional regulation
Fos-like antigen 2 (Fosl2)	NM_008037.3	**+ 3.00**	**0.000**	**+ 1.69**	**0.032**	Regulation of developmental processes
v-maf musculoaponeurotic fibrosarcoma oncogene family, protein F (avian) (Maff)	NM_010755.2	**+ 2.58**	**NS**	**+ 2.72**	**0.015**	Regulation of acute-phase reaction
Activating transcription factor 3 (Atf3)	NM_007498.2	**+ 1.54**	**NS**	**+ 2.69**	**0.039**	Regulator of cell proliferation, differentiation, and transformation
Kruppel-like factor 2 (lung) (Klf2)	NM_008452.1	**+ 2.44**	**0.027**	**+ 1.08**	**NS**	Inhibition of cell proliferation
T-box 3 (Tbx3), transcript variant 2	NM_011535.2	**+ 1.29**	**NS**	**+ 1.98**	**0.003**	Possible cellular stress response
LPS-induced TN factor (Litaf)	NM_019980.1	**+ 2.76**	**NS**	**+ 1.87**	**0.037**	Cytokine signaling
**Histones**						
Histone 1, H2ai (Hist1h2ai)	NM_178182	**- 2.07**	**NS**	**- 2.81**	**0.038**	Chromatin structure
Histone 1, H2ao (Hist1h2ao)	NM_178185.1	**- 2.07**	**NS**	**- 2.62**	**0.048**	Chromatin structure
						
**Oxidative stress**						
Metallothionein (Mt1)	NM_013602.2	**+ 3.01**	**NS**	**+ 12.18**	**0.003**	Removal of oxidant radicals
Sulfiredoxin 1 homolog (Npn3)	NM_029688.2	**+ 1.68**	**NS**	**+ 10.61**	**0.005**	Oxidant reduction
Metallothionein 2 (Mt2)	NM_008630.1	**+ 2.53**	**NS**	**+ 9.53**	**0.008**	Removal of oxidant radicals
						
**Angiogenesis**						
ADAM metallopeptidase with thrombospondin type 1 motif 9 (Adamts9)	XM_204236.1	**+ 1.76**	**NS**	**+ 2.80**	**0.010**	Inhibition of angiogenesis
EGL nine homolog 3 (C. elegans) (Egln3)	NM_028133.1	**+ 1.35**	**NS**	**+ 2.22**	**0.042**	Hypoxia Response
Platelet derived growth factor alpha (Pdgfa)	NM_008808	**- 1.07**	**NS**	**- 1.92**	**0.009**	Activation of angiogenesis
						
**Solute carriers**						
Short calcium-binding mitochondrial carrier 2 (Slc25a25)	NM_146118.2	**+ 2.98**	**0.016**	**+ 3.01**	**NS**	Calcium-dependent mitochondrial solute carrier
Tweety homolog 1 (Drosophila) (Ttyh1)	NM_021324.3	**+ 1.34**	**NS**	**+ 2.16**	**0.027**	Chloride anion channel
Sodium-dependent vitamin C transporter 2 (Slc23a2)	NM_018824.2	**+ 1.07**	**NS**	**+ 1.99**	**0.021**	Vitamin C transport
Solute carrier family 10 (sodium/bile acid cotransporter family), member 3 (Slc10a3)	NM_145406.1	**+ 1.30**	**NS**	**+ 1.93**	**0.015**	Organic anion/sodium transport?
solute carrier family 20, member 1 (Slc20a1)	NM_015747.1	**+ 1.81**	**0.008**	**+ 1.51**	**NS**	Phosphate transporter
						
**Apoptosis and cell cycle**						
Polo-like kinase 3 (Plk3)	NM_013807.1	**+ 2.39**	**NS**	**+ 3.52**	**0.009**	Regulation of cell cycle
Cyclin-dependent kinase inhibitor 1C (Cdkn1c)	NM_009876.2	**- 2.17**	**NS**	**- 3.45**	**0.005**	Apoptosis
Cyclin-dependent kinase inhibitor 1A (Cdkn1a)	NM_007669.2	**+ 2.28**	**0.047**	**+ 3.04**	**0.019**	Apoptosis
S100 calcium binding protein A8 (calgranulin A) (S100a8)	NM_013650.1	**- 1.04**	**NS**	**+ 2.81**	**0.039**	Cell cycle
DNA-damage-inducible transcript 4-like (Ddit4l)	NM_030143.2	**- 1.04**	**NS**	**- 2.60**	**0.032**	Apoptosis
Cytokine induced apoptosis inhibitor 1 (Ciapin1)	NM_134141.2	**+ 1.21**	**NS**	**+ 2.03**	**0.016**	Cytokine-induced inhibitor of apoptosis
Lectin, galactose binding, soluble 3 (Lgals3)	NM_010705.1	**- 1.29**	**NS**	**+ 1.98**	**0.019**	Possible cell cycle regulator
						
**Cancer and DNA repair**						
Jun-B oncogene (Junb)	NM_008416.1	**+ 5.20**	**0.021**	**+ 1.81**	**NS**	Cell signaling
AXIN1 up-regulated 1 (Axud1)	NM_153287.2	**+ 2.84**	**0.040**	**+ 1.49**	**NS**	Tumor suppressor function?
Growth arrest and DNA-damage-inducible 45 alpha (Gadd45a)	NM_007836.1	**- 1.01**	**NS**	**+ 2.03**	**0.027**	Induced by DNA damage
Excision repair cross-complementing rodent repair deficiency, complementation 5 (Ercc5)	NM_011729	**+ 1.51**	**0.050**	**+ 1.93**	**0.026**	Repair of UV-induced DNA damage
						
**Ubiquitin-proteasome system**						
Ubiquitin G (Ubg)	N/A	**+ 1.30**	**NS**	**+ 2.36**	**0.003**	Ubiquitin-proteasome system
Ubiquitin C (Ubc)	XM_147315.1	**+ 1.30**	**NS**	**+ 2.24**	**0.002**	Ubiquitin-proteasome system
F-box only protein 34 (Fbxo34)	NM_030236.1	**+ 1.17**	**NS**	**+ 2.12**	**0.012**	Ubiquitin-proteasome system
Ubiquitin specific protease 2 (Usp2), transcript variant 2	NM_198091.1	**- 1.24**	**NS**	**+ 2.08**	**0.032**	Ubiquitin-proteasome system
						
**Miscellaneous genes**						
Midnolin (Midn)	NM_021565.1	**+ 2.53**	**NS**	**+ 4.87**	**0.002**	Neurogenesis
Imprinted and ancient (Impact)	NM_008378.1	**+ 2.04**	**NS**	**+ 4.70**	**0.005**	Unknown
Emerin (Emd)	NM_007927.1	**+ 1.82**	**NS**	**+ 4.07**	**0.005**	Nuclear envelope regulation?
Downstream of Stk11 (Dos)	XM_125771	**+ 2.25**	**NS**	**+ 3.83**	**0.010**	Unknown
Phosphodiesterase 4D (Pde4d)	NM_011056.1	**+ 2.06**	**0.010**	**+ 3.78**	**0.014**	Regulation of cAMP levels
Interferon gamma inducible protein 30 (Ifi30)	NM_023065.2	**+ 1.31**	**NS**	**+ 3.23**	**0.019**	Antigen processing
Y box protein 3 (Ybx3)	AK029441	**+ 2.59**	**0.047**	**+ 2.91**	**0.010**	Unknown
DNA segment, Chr 19, Wayne State University 162, expressed (D19Wsu162e)	NM_146099	**+ 1.38**	**NS**	**+ 2.85**	**0.006**	Unknown
Alpha-kinase 2 (Alpk2)	XM_128981	**+ 1.16**	**NS**	**+ 2.82**	**0.005**	Amino acid phosphorylation
Tumor necrosis factor receptor superfamily, member 12a (Tnfrsf12a)	NM_013749.1	**+ 2.11**	**NS**	**+ 2.76**	**0.014**	Unknown
Synaptopodin 2-like (Synpo2I)	NM_175132	**- 1.12**	**NS**	**- 2.70**	**0.005**	May modulate actin shape
Alkaline phosphatase 2 (Akp2)	NM_007431.1	**+ 1.59**	**0.015**	**+ 2.69**	**0.028**	Phosphatase
Syndecan 4 (Sdc4)	NM_011521.1	**+ 1.55**	**NS**	**+ 2.68**	**0.005**	Intracellular signaling receptor
Alpha Tubulin 6 (Tuba6)	XM_147357.1	**+ 1.14**	**NS**	**+ 2.58**	**0.025**	Microtubules formation
Avian musculoaponeurotic fibrosarcoma (v-maf) AS42 oncogene homolog (Maf)	N/A	**+ 1.93**	**0.040**	**+ 2.51**	**0.008**	Unknown
Glutamic pyruvate transaminase (alanine aminotransferase) 2 (Gpt2)	NM_173866.1	**+ 1.13**	**NS**	**+ 2.04**	**0.032**	Amino acid metabolism
Thrombomodulin (Thbd)	NM_009378.1	**+ 1.87**	**0.013**	**+ 2.02**	**NS**	Anticoagulant pathway
CCR4 carbon catabolite repression 4-like (S. cerevisiae) (Ccrn4l)	NM_009834.1	**+ 1.11**	**NS**	**+ 2.01**	**0.041**	Predicted transcription factor
Small chemokine (C-C motif) ligand 11 (Ccl11)	NM_011330.1	**+ 1.26**	**NS**	**+ 1.96**	**0.010**	Cytokine signaling
Mitogen-activated protein kinase kinase kinase 6 (Map3k6)	NM_016693	**+ 1.13**	**NS**	**+ 1.94**	**0.036**	MAPK signaling pathway
Chemokine (C-C motif) ligand 9 (Ccl9)	NM_011338.2	**+ 1.01**	**NS**	**+ 1.91**	**0.018**	Cytokine signaling
Microtubule-associated protein 1 light chain 3 alpha (Map1lc3a)	NM_025735.1	**+ 1.23**	**NS**	**+ 1.90**	**0.016**	Mediates interactions between microtubules and cytoskeleton
MAP kinase-interacting serine/threonine kinase 2 (Mknk2)	NM_021462.2	**+ 1.27**	**NS**	**+ 1.87**	**0.017**	MAPK signaling pathway
Lymphocyte antigen 6 complex, locus A (Ly6a)	NM_010738.2	**- 1.14**	**NS**	**+ 1.87**	**0.018**	Cell adhesion and cell signaling
Ssemaphorin 3F (Sema3f)	NM_011349.2	**+ 1.47**	**NS**	**+ 1.86**	**0.046**	Cell signaling?
Zinc finger protein 46 (Zfp46)	NM_009557.1	**- 1.41**	**NS**	**- 1.86**	**0.005**	Unknown
Optineurin (Optn)	NM_181848.3	**+ 1.20**	**NS**	**+ 1.85**	**0.016**	Possible mediator of apoptosis?
Vasodilator-stimulated phosphoprotein (Vasp)	NM_009499	**+ 1.12**	**NS**	**- 1.85**	**0.027**	Focal adhesion stability

**Table 2:** Significant differentially expressed non-annotated genes in skeletal muscle after acute systemic administration of β_2_-AR agonist

**Gene Name**	**Genebank ID**	**1 HOUR**		**4 HOURS**	
			
		**change**	**p-value**	**change**	**p-value**
RIKEN cDNA 3300001A09	XM_134869.3	**+ 4.68**	**NS**	**+ 5.32**	**0.042**
RIKEN cDNA 1200016E24	XM_489305	**+ 4.90**	**0.016**	**+ 2.32**	**NS**
cDNA sequence BC036718	NM_153136.1	**+ 1.81**	**NS**	**+ 3.96**	**0.004**
RIKEN cDNA C330006P03	N/A	**+ 1.11**	**NS**	**+ 3.78**	**0.006**
RIKEN cDNA B430214A04	NM_146018.1	**+ 1.42**	**NS**	**+ 3.05**	**0.004**
RIKEN cDNA 6430548M08	NM_172286	**+ 1.15**	**NS**	**+ 2.94**	**0.025**
RIKEN cDNA A830030H10	AK080627	**+ 1.96**	**NS**	**+ 2.69**	**0.025**
RIKEN cDNA 4833406M21	N/A	**+ 1.38**	**NS**	**+ 2.66**	**0.004**
RIKEN cDNA 1300002F13	NM_133753.1	**+ 2.54**	**0.013**	**+ 1.11**	**NS**
RIKEN cDNA 9530083O12	N/A	**+ 2.53**	**0.013**	**+ 1.69**	**NS**
RIKEN cDNA 1200015N20	NM_024244.3	**- 1.06**	**NS**	**+ 2.52**	**0.010**
Hypothetical protein 4933408F15	NM_172715.1	**+ 1.24**	**NS**	**+ 2.52**	**0.010**
RIKEN cDNA A730009E18	N/A	**+ 1.34**	**NS**	**+ 2.42**	**0.005**
RIKEN cDNA A430107N12	N/A	**+ 1.04**	**NS**	**+ 2.21**	**0.036**
RIKEN cDNA 5830446M03	NM_133934.2	**+ 1.32**	**NS**	**+ 2.14**	**0.016**
Similar to heart alpha-kinase (LOC381181)	XM_355107.1	**+ 1.08**	**NS**	**+ 2.11**	**0.009**
RIKEN cDNA 2810426P10	XM_148728.1	**+ 1.03**	**NS**	**+ 2.08**	**0.008**
Weakly similar to protein transport protein SEC24A (SEC24-RELATED PROTEIN A)	AK038836	**+ 2.07**	**0.041**	**+ 1.29**	**NS**
RIKEN cDNA 2310040A07	N/A	**+ 1.06**	**NS**	**+ 2.07**	**0.047**
RIKEN cDNA 8030450I18	N/A	**+ 1.33**	**NS**	**+ 2.05**	**0.043**
RIKEN cDNA 3021401C12	N/A	**+ 1.17**	**NS**	**+ 1.98**	**0.015**
RIKEN cDNA 2900078C09	N/A	**+ 1.46**	**NS**	**+ 1.95**	**0.039**
RIKEN cDNA 1110033I14	XM_126635.1	**+ 1.72**	**NS**	**+ 1.95**	**0.044**
cDNA sequence BC023105	NM_145357	**- 1.07**	**NS**	**- 1.92**	**0.039**

### Functional categorization of genes differentially regulated by β_2_-AR activation

Genes presented in table 1 were grouped according to their potential relevant function in skeletal muscle. The potential relevant function is based on the authors' opinion gained from a combination of Illumina Gene Ontology classifications, Ingenuity Pathway Analysis , Online Mendelian Inheritance in Man (OMIM; ), and AceView  searches. The Illumina BeadArray expression analysis revealed significant changes in the expression of genes in several functional categories at 1 and 4 hours following formoterol administration, including genes involved in skeletal muscle hypertrophy/growth, myoblast differentiation, metabolism, circadian rhythm, transcription, histones, oxidative stress, angiogenesis, solute carriers, apoptosis, cell cycle, cancer, DNA repair, and the ubiquitin-proteasome system.

### Validation of differential gene expression by quantitative RT-PCR

**Figure 1:**
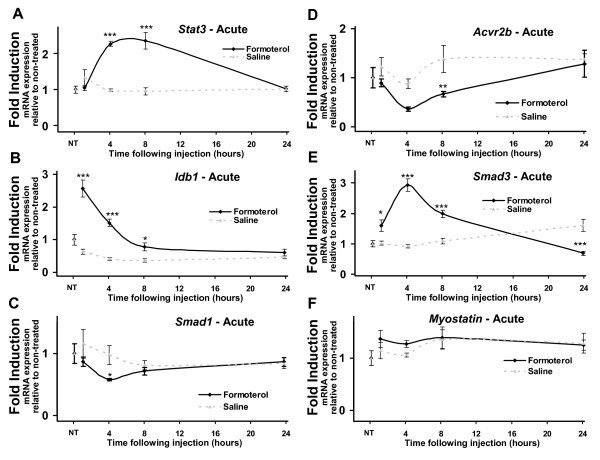
**Acute systemic administration of formoterol alters the expression of genes associated with muscle growth and differentiation at multiple timepoints**. Quantitative RT-PCR was used to assay the expression of ***A***. *Stat3*, ***B***. *Idb1*, ***C***. *Smad1*, ***D***. *Acvr2b*, ***E***. *Smad3*, and ***F***.*Myostatin *mRNAs in tibialis anterior over acute timepoints. Muscles were removed at 1, 4, 8 and 24 hours following a single intraperitoneal injection of formoterol or saline vehicle (NT = no treatment). Results were normalized against 36B4 at each timepoint. Statistical significance was assessed using a one-way ANOVA with Bonferroni's post-test where p < 0.05 (*), p < 0.01 (**) and p < 0.001 (***). Unmarked data points are non-significant.

**Figure 2:**
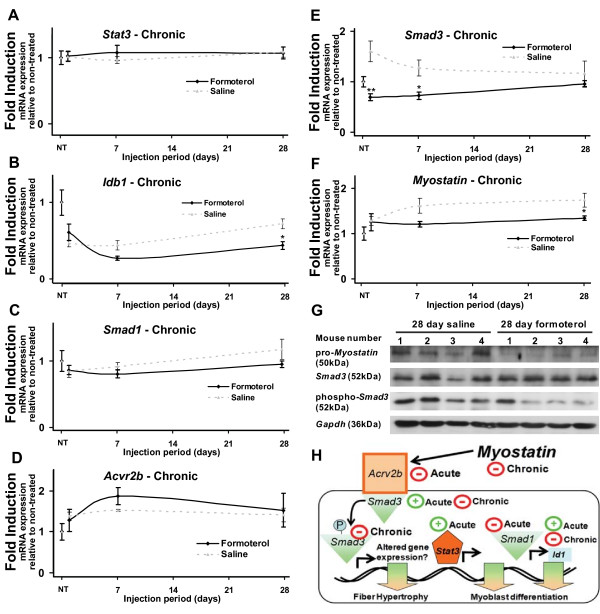
**Chronic systemic administration of formoterol alters the expression of genes associated with skeletal muscle hypertrophy and myogenesis at multiple timepoints**. Quantitative RT-PCR was used to assay the expression of ***A***. *Stat3*, ***B***. *Idb1*, ***C***. *Smad1*, ***D***. *Acvr2b*, ***E***. *Smad3*, and ***F***.*Myostatin *mRNAs in tibialis anterior over chronic timepoints. Muscles were removed at 1, 7 and 28 days following daily intraperitoneal injection of formoterol or saline vehicle (NT = no treatment). Results were normalized against 36B4 at each timepoint. Data are expressed as mean ± SEM (n = 5). Statistical significance was assessed using a one-way ANOVA with Bonferroni's post-test where p < 0.05 (*), p < 0.01 (**) and p < 0.001 (***). Unmarked data points are non-significant. ***G***. Protein levels of *Myostatin *precursor (pro-*Myostatin*), *Smad3*, phosphorylated *Smad3*, and *Gapdh *were visualized by Western blotting performed on tibialis anterior muscle following 28 days of formoterol/saline administration in four animals for each treatment. ***H***. Diagrammatic representation of acute and chronic gene expression changes related to skeletal muscle hypertrophy and myogenesis in response to formoterol.

**Figure 3:**
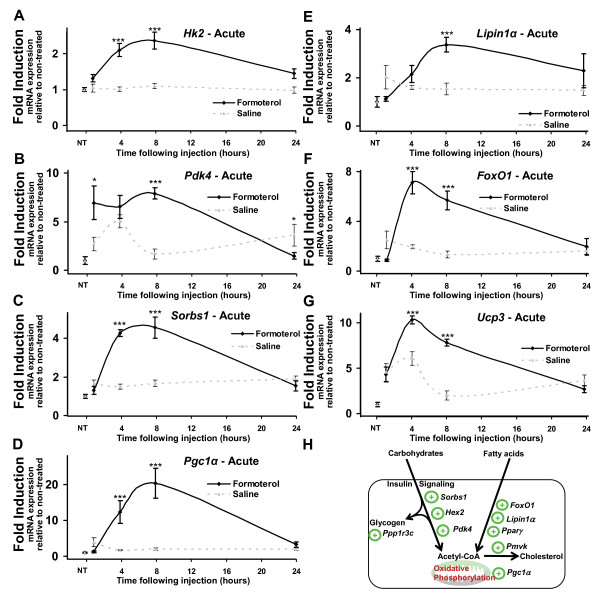
**Acute systemic administration of formoterol alters the expression of genes associated with metabolism**. Quantitative RT-PCR was used to assay the expression of ***A***. *Hk2*, ***B***. *Pdk4*, ***C***. *Sorbs1*, ***D***. *Pgc1α*, ***E***.*Lipin1α*, ***F***. *FoxO1, and ****G***.*Ucp3 *mRNAs in tibialis anterior over acute timepoints. Muscles were removed at 1, 4, 8 and 24 hours following a single intraperitoneal injection of formoterol or saline vehicle (NT = no treatment). Results were normalized against 36B4 at each timepoint. Statistical significance was assessed using a one-way ANOVA with Bonferroni's post-test where p < 0.05 (*), p < 0.01 (**) and p < 0.001 (***). Unmarked data points are non-significant. ***G***. Diagrammatic representation of acute gene expression changes related to metabolic in skeletal muscle in response to formoterol.

**Figure 4:**
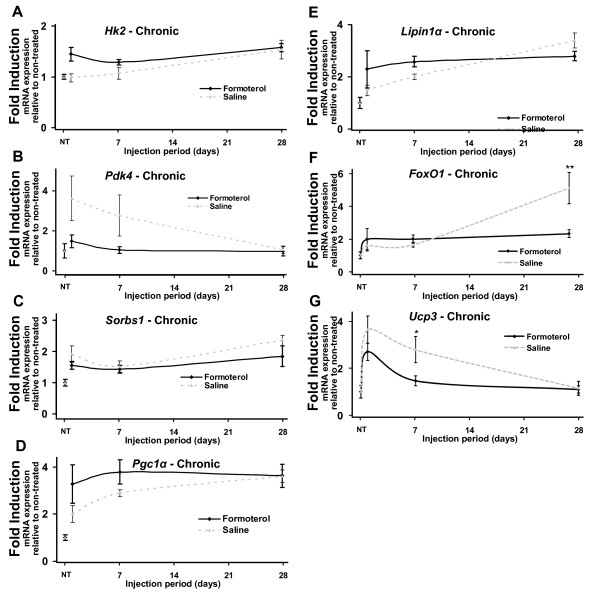
**Chronic systemic administration of formoterol alters the expression of genes associated with metabolism**. Quantitative RT-PCR was used to assay the expression of ***A***. *Hk2*, ***B***. *Pdk4*, ***C***.* Sorbs1*, ***D***. *Pgc1α*, ***E***. *Lipin1α*, ***F***. *FoxO1*, *and ****G***.*Ucp3 *mRNAs in tibialis anterior over chronic timepoints. Muscles were removed at 1, 7 and 28 days following daily intraperitoneal injection of formoterol or saline vehicle (NT = no treatment). Results were normalized against 36B4 at each timepoint. Statistical significance was assessed using a one-way ANOVA with Bonferroni's post-test where p < 0.05 (*), p < 0.01 (**) and p < 0.001 (***). Unmarked data points are non-significant.

**Figure 5:**
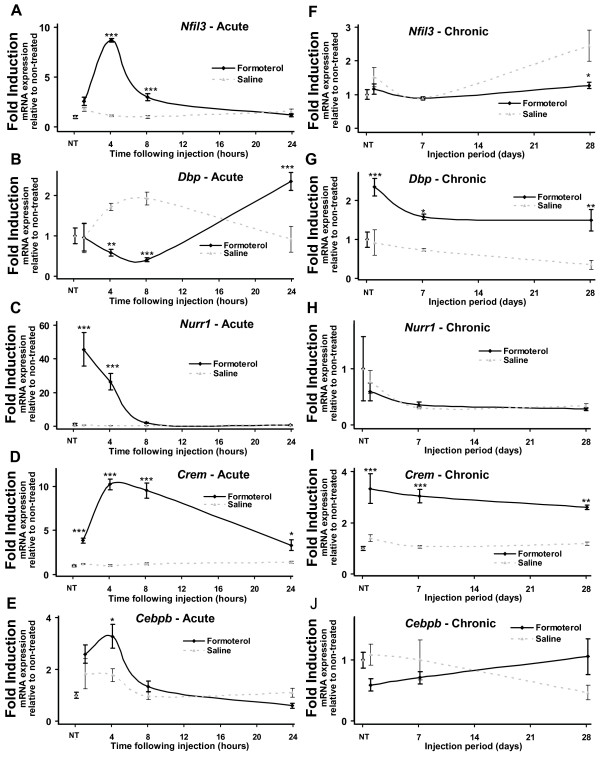
**Acute and chronic systemic administration of formoterol alters the expression of genes associated with circadian rhythm and transcriptional regulation**. Quantitative RT-PCR was used to assay the expression of ***A***. *Nfil3*, ***B***. *Dbp*, ***C***. *Nurr1*, ***D***. *Creb*, and ***E***.*Cebpb *mRNAs in tibialis anterior over acute timepoints. Muscles were removed at 1, 4, 8 and 24 hours following a single intraperitoneal injection of formoterol or saline vehicle (NT = no treatment). For chronic timepoints, the expression of ***F***. *Nfil3*, ***G***. *Dbp*, ***H***. *Nurr1*, ***I***. *Creb*, and ***J***.*Cebpb *mRNAs were measured in tibialis anterior removed at 1, 7 and 28 days following daily intraperitoneal injection of formoterol or saline vehicle (NT = no treatment). All results were normalized against 36B4 at each timepoint. Data are expressed as mean ± SEM (n = 5). Statistical significance was assessed using a one-way ANOVA with Bonferroni's post-test where p < 0.05 (*), p < 0.01 (**) and p < 0.001 (***). Unmarked data points are non-significant.

The expression of 16 genes (*Stat3, Idb1, Smad1, Smad3, Hk2, Pdk4, Sorbs1, Pgc1α, Lipin1α, FoxO1, Ucp3, Nfil3, Dbp, Nurr1, Crem, and Cebpb*) that were identified as differentially expressed by Illumina beadarray analysis (Table 1) and associated with the regulation of skeletal muscle mass, circadian rhythm and metabolism were validated and examined in greater detail following acute (1-24 h) and chronic (1-28 days) formoterol administration via quantitative RT-PCR (qRT-PCR; Figures 1, 2, 3, 4 and 5). All qRT-PCR analyses were performed on an independent/different set of formoterol treated mice (n = 5 per timepoint) than the group used in the Illumina BeadArray study. All 16 gene analyzed by qRT-PCR on independent animals closely mirrored the Illumina changes at both timepoints, highlighting the robust nature of the Illumina platform.

### Formoterol administration alters the expression of genes associated with skeletal muscle hypertrophy and differentiation: attenuation of myostatin signaling

Several differentially expressed genes associated with the regulation of muscle differentiation and mass (identified from the Illumina BeadArray) were examined over an acute and chronic time course of formoterol administration using qRT-PCR. In addition we also examined the expression of myostatin and the myostatin receptor, activin receptor IIB (*Acvr2b*) that are critical modulators of muscle mass. We examined these genes using qRT-PCR as *Acvr2b *was down-regulated at 4 hour in the Illumina BeadArray, however it did not pass statistical analysis (data not shown). Tibialis anterior muscle was isolated from groups (n = 5) of male mice, treated with either the specific β_2_-AR agonist treatment or saline (vehicle control) and assayed at 0, 1, 4, 8 and 24 h post treatment. Significant changes in expression at one or more timepoints were observed in the mRNAs encoding signal transducer and activator of transcription 3 (*Stat3*; Figure 1A), inhibitor of DNA binding 1 (*Idb1*; Figure 1B), small mothers against decapentaplegic homolog 1 (*Smad1*; Figure 1C), *Acvr2b *(Figure 1D), and small mothers against decapentaplegic homolog 3 (*Smad3*; Figure 1E). We did not observe any significant changes in myostatin expression, after acute β_2_-AR agonist treatment (Figure 1F)

To examine chronic changes induced by formoterol administration, qRT-PCR was used to examine expression of genes from chronically treated mice (after 1, 7 and 28 days of agonist treatment). Similar to acute timepoints, tibialis anterior was isolated from groups (n = 5) of male mice, treated daily with either the specific β_2_-AR agonist formoterol or saline (vehicle control) and assayed at 0, 1, 7 and 28 days of treatment. Chronic formoterol administration was associated with a significant attenuation in the expression of the mRNAs encoding *Idb1 *(Figure 2B), *Smad3 *(Figure 2E) and myostatin (Figure 2F) after 7 or 28 days. No significant changes were observed following chronic formoterol administration in the expression *of the mRNAs encoding Stat3 *(Figure 2A), *Smad1 *(Figure 2C), and *Acvr2b *(Figure 2D), despite significant repression following acute formoterol administration.

To examine the effect of chronic β_2_-AR agonist treatment on critical regulators of the myostatin signaling pathway we examined the levels of the Myostatin precursor (pro-Myostatin), *Smad3*, phosphorylated *Smad3 *relative to *Gapdh*. We assayed levels by Western blotting analysis of tibialis anterior muscle (contralateral to muscle used for qRT-PCR analysis) following 28 days of formoterol/saline administration in four animals for each treatment (Figure 2G). Consistent with the qRT-PCR data, at the protein level, pro-Myostatin appears subtley (but consistently) suppressed following 28 days of formoterol administration. In concordance, the levels of *Smad3 *phosphorylation following the chronic formoterol administration are also reduced (in 3 out of 4 mice), while total *Smad3 *appears unchanged.

In summary, β_2_-adrenergic stimulation mediates changes in the expression of several genes associated with myostatin signaling, and the regulation of muscle mass (Figure 2H).

### Formoterol administration alters the expression of genes associated with metabolism and circadian rhythm

Many genes that regulate and/or are directly involved in metabolism are regulated in a circadian manner. The Illumina BeadArray study identified differential expression of several genes involved in these pathways. Consequently, we utilized qRT-PCR to validate the differential expression of these genes after acute or chronic administration of fomoterol (vs vehicle) in tibialis anterior as detailed above. Significant expression changes at one or more timepoints were observed in several genes involved in metabolism, including, hexokinase 2 (*Hk2*; Figure 3A), pyruvate dehydrogenase kinase 4 (*Pdk4*; Figure 3B), sorbin and SH3 domain containing 1 (*Sorbs1*; Figure 3C), PPARγ coactivator 1 alpha (*Pgc1α*; Figure 3D), *Lipin1α *(Figure 3E); forkhead box O1 (*FoxO1*; Figure 3F), and uncoupling protein 3 (*Ucp3*; Figure 3G). In the context of crosstalk between β-AR signaling and *Nor-1 *(NR4A3) signaling in skeletal muscle, we have previously identified and examined the induction of *Pdk4*, *Pgc1α, FoxO1*, and *Lipin1α *over following acute β-AR activation [28,36].

Chronic formoterol administration significantly altered the expression of *FoxO1 *(Figure 4F) and *Ucp3 *(Figure 4G) at 7 and 28 days respectively, while *Hk2 *(Figure 4A), *Pdk4 *(Figure 4B), *Sorbs1 *(Figure 4C), *Pgc1α *(Figure 4D), and *Lipin1α *(Figure 4E) were not significantly altered.

The expression of two peripheral tissue regulators of circadian rhythm, albumin D-box binding protein (*Dbp*), and nuclear factor interleukin 3 regulated (*Nfil3*) were significantly dysregulated by both acute and chronic formoterol administration (Figures 5A, B, F and 5G).

In summary, formoterol administration mediated the significant modulates of several metabolic genes (for example *Pgc1α*, *Lipin1α*, *Pdk4*, *FoxO1*, *Hk2*, *Ucp3*, *Sorbs1 *etc) associated with the transient induction of oxidative metabolism, particularly following acute stimulation of β-AR's. Interestingly, the expression of these genes was normalized 24 h post treatment, and remained at control levels throughout the 28 days of formoterol administration. In addition, acute and chronic β_2_-AR agonist treatment significantly regulates the expression of two critical regulators of circadian cycling.

### Altered transcriptional regulation following formoterol administration

We have previously demonstrated that β-AR agonists markedly increased the expression of the NR4A subgroup (*Nur77*, *Nurr1 *and *Nor-1*) of nuclear receptor transcription factors in skeletal muscle [27-29]. From the Illumina BeadArray, ten transcription factors (not placed in other categories) were induced by formoterol 1 and 4 h post-administration (Table 1). At one or more timepoints, acute administration of formoterol significantly induced nuclear receptor related 1 protein (*Nurr1*; Figure 5C), cAMP responsive element modulator (*Crem*; Figure 5D), and CCAAT/enhancer binding protein β (*Cebpb*; Figure 5E). In contrast, *Crem *was the only transcription factor to remain elevated throughout the 28 day formoterol administration period (Figure 5I compared to Figures 5H and 5J).

## Discussion

A variety of studies have demonstrated that acute (and chronic) β-AR stimulation in skeletal muscle induces hypertrophy, and modulates oxidative metabolism, mitochondrial parameters, energy expenditure, lipolysis [1,16-20], glucose transport [37], and glucose oxidation [20].

To examine gene expression associated with these effects, we utilized Illumina BeadArray gene expression profiling to examine global gene expression in skeletal muscle in response to acute systemic administration (1 and 4 hours) of a specific β_2_-AR agonist (formoterol). In this study we have revealed that β_2_-AR agonist treatment altered the expression of several genes associated with myostatin signaling, a previously unreported effect of β-AR signaling in skeletal muscle. This is also the first study to demonstrate a β_2_-AR agonist regulation of circadian rhythm genes, indicating crosstalk between β-AR signaling and circadian cycling in skeletal muscle

### Skeletal muscle hypertrophy and myoblast differentiation

Previous studies have demonstrated that systemic administration of β_2_-AR agonists induces hypertrophy in both skeletal and cardiac muscle [1-3,38]. Furthermore, β_2_-AR agonists have been found to prevent or reverse the muscle wasting and weakness associated with numerous conditions [for review see Ryall & Lynch, 2008 [15]]. However, the cellular and molecular mechanisms underlying these changes have yet to be fully elucidated. The scientific literature is not consistent on whether β-AR agonists increase myofibrilar protein mass during hypertrophy by increased protein biosynthesis, decreased proteolysis, or both.

Reeds et al. [4] suggested that β-AR agonists do not increase global protein biosynthesis, however recent reports suggested that β-AR agonists increase the expression of several contractile proteins [30,39,40], myogenin [41], and initiators of protein translation [30]. In addition to effects on protein synthesis a number of studies have attributed increased myofibrilar protein content to an inhibition of myofibrilar proteolysis [4,8,42], possibly via inhibition of ubiquitin-proteasome mediated degradation [8,10,11], Ca^2+^-dependent proteolysis [12] or calpain-mediated proteolysis [6,13,14].

At the molecular level, the gene ankyrin repeat and SOCS box protein *Asb15*, known to promote protein synthesis and myoblast differentiation, has been found to be induced by β-AR stimulation [43-46]. Similarly, both *Igf1 *and *Igf2 *mRNA expression has been observed to increase following chronic systemic administration of β-AR agonists, suggesting the involvement of these growth factors in hypertrophy [40,47]. Interestingly, in the current study no significant acute changes were observed in *Asb15 *or *Igf1 *(data not shown), however *Igf2 *expression was down-regulated (non-significantly; data not shown) after four hours, a result that is not consistent with these previous studies [40,47].

We have also included a more detailed table (Table 3), which compares the Illumina BeadArray information in this study to previously published hypotheses on β-AR agonist-induced hypertrophy.

**Table 3:** Summary of previous reports describing the known molecular effects of β-AR agonist-induced hypertrophy in skeletal muscle

**β-AR agonist-induced hypertrophy hypothesis**	**Reference(s)**	**Comparison to our study**
Increased expression of skeletal muscle contractile proteins	[30,39,40]	No significant changes were observed in contractile proteins at 1 and 4 hours in our Illumina array data. In the previous referenced studies, contractile proteins were only examined following chronic β-AR agonist administration. Increased myosin heavy chain was observed at protein level [39], and may not be present at mRNA level.
		
Increased mRNA expression of myogenin, a key developmental regulator of functional skeletal muscle	[30]	No significant changes were observed in myogenin at 1 and 4 hours in our Illumina array data. In Spurlock et al. [30], increased myogenin mRNA expression was only observed at 24 hours following β-AR agonist administration. Our earlier timepoints may miss this change.
		
Increased expression of initiators of protein translation	[30]	Our Illumina array data showed no significant changes in any genes that are known initiators of protein translation. In Spurlock et al. [30], increased expression of mRNA encoding initiators of protein translation were observed mainly at 24 hours following β-AR agonist administration. Our earlier timepoints may miss these changes.
		
Decreased myofibrilar proteolysis via inhibition of the ATP-ubiquitin-dependent proteolytic system	[8,10,11]	We observed the induction of four genes associated with ubiquitin-proteolytic system (Ubg, Ubc, Fbxo34 and Usp2). This is in contrast to the referenced studies that demonstrated inhibition of the ATP-ubiquitin-dependent proteolytic system via chronic β-AR agonist administration on skeletal muscle. The induction we observed may represent a mechanism whereby acute β-AR signaling induces proteolysis for myofibril repair following exercise (which is known to induce β-AR signaling [75]).
		
Decreased myofibrilar proteolysis via Ca^2+^-dependent or calpain-mediated proteolysis	[12]	Our Illumina array data showed no significant changes in any genes associated with calpain-mediated or other Ca^2+^-dependent proteolytic genes.
		
Decreased expression of SOCS box protein *Asb15, which is a negative regulator *of protein synthesis and myoblast differentiation	[43-46]	We did not observe repression (or induction) of *Asb15 *mRNA in the Illumina array data at either timepoint. In the referenced studies, *Asb15 is repressed at 12-24 hours*. Our earlier timepoints may miss this expression change, however there is the potential for a transcription factor listed in our study to repress *Asb15*.
		
Induction of *Igf1 *mRNA expression	[30]	We did not observe any induction of *Igf1 *in the Illumina array data at either timepoint. In Spurlock et al. [30], increased expression of Igf1 was observed mainly at 24 hours post β-AR agonist administration.
		
Induction of *Igf2 *mRNA expression	[40,47]	*Igf2 *was repressed 2.61 fold, however this was removed due to multiple testing correction. Although this result is not consistent with the referenced previous studies, *Igf2 *mRNA in these previous studies was only examined after chronic β-AR agonist administration.

In our study, Illumina BeadArray expression profiling analysis revealed several gene expression changes associated with the regulation of skeletal muscle mass and myoblast differentiation. From the array (and qRT-PCR), we observed alterations in *Stat3*, and *Smad3, Acvr2b *three genes directly associated with the regulation of muscle hypertrophy. Both *Acvr2b *(a key myostatin receptor) and *Smad3 *are downstream mediators of myostatin, a well-characterized negative regulator of muscle mass. With qRT-PCR, we also observed a subtle, but significant attenuation of the mRNAs encoding myostatin in response to chronic β_2_-AR agonist treatment, however this was not detected by the acute Illumina analysis. This is concordant with the chronic effects of formoterol administration on skeletal muscle, and could provide a partial mechanistic basis for hypertrophy.

While muscle growth from β-AR agonists is associated with hypertrophy, enhancement of myogenesis could lead to proliferation, differentiation, and/or recruitment of satellite cells into muscle fibers to promote muscle growth. We observed significant changes in *Itgb1bp3, Smad1, Smad3, FoxO1 *(listed under metabolism in Table 1) and, *Idb1*, genes believed to play an important role in regulating myogenesis. We observed significant changes in *Smad1 *expression, and while closely related to *Smad3*, *Smad1 *has not been associated with skeletal muscle hypertrophy, however it may have a role in the regulation of myogenesis [48]. A complex of *Smad1 *and *4 *has been shown to transactivate *Id1 *[49], another myogenic gene altered by formoterol administration. Interestingly, we observed a significant repression of *FoxO1*, a negative regulator of myogenesis, following 28 days of formoterol administration [50-52], thus suggesting β_2_-AR agonist enhance myogenesis in the context of chronic treatment. Furthermore, myostatin may also be a transcriptionaly regulated by *FoxO1 *[53], possibly suggesting coordinate regulation of hypertrophy and myogenesis via multiple mechanisms including *Fox01*/myostatin signaling. Moreover, we have performed Western (immunoblot) analysis on the tibialis anterior muscle from 28 day vehicle (saline) and β_2_-AR agonist treatment (formoterol) treated mice (n = 4 mice per treatment). This demonstrated that formoterol treatment suppressed expression of pro-Myostatin, and the levels of phospho-*Smad3*. These observations are in concordance with the attenuation of the myostatin signaling pathway and therefore the hypertrophic phenotype [54,55].

In summary, formoterol administration induced significant changes in genes associated with skeletal muscle hypertrophy and myogenesis. A broad overview of these expression changes are provided in Figure 2H, notably highlighting possible crosstalk between β-AR signaling and myostatin/*Smad3 *signaling pathway.

### Metabolism

Many previous studies have implicated β-AR signaling in the control of metabolism. Mice lacking all three β-AR are unable to effectively regulate energy expenditure and thus develop obesity on a high-fat diet [25]. In terms of skeletal muscle, β-AR agonist administration has been found to modulate oxidative metabolism, energy expenditure, lipolysis [1,16-20], glucose transport [22] glucose oxidation [20] and mitochondrial morphology [56]. In the current study we found that formoterol administration significantly induced the expression of 14 genes associated with metabolism and mitochondrial function greater than 1.85 fold (Table 1). These consisted of genes involved in lipid regulation and metabolism, including *Lipin1α*, *FoxO1*, scavenger receptor class B member 1 (*Scarb1*), phosphomevalonate kinase (*Pmvk*), plasma membrane associated protein (*S3-12*), peroxisome proliferator-activated receptor γ (*Pparγ*), and *Ucp3*. The largest change observed was the induction of *Pgc-1α*, a key transcriptional regulator of oxidative metabolism and regulator of skeletal muscle fiber type and therefore lipid metabolism. Genes involved in glucose metabolism/storage and insulin signaling were up-regulated, *Pdk4*, protein phosphatase 1 regulatory subunit 3C (*Ppp1r3c*), and *Hk2*.

Several of these genes have been previously identified in our studies following *in vitro *and *in vivo *treatment of muscle with a β_2_-AR agonist. For example, the induction of the mRNAs encoding *Pgc1α*, *Lipin1α, Pdk4 *and *FoxO1 *in skeletal muscle were identified in our study [28] and that of Miura et al. [36].

Interestingly, the expression levels of *Pgc1α*, *Lipin1 *and *FoxO1 *have also been found to be increased following exercise [57], suggesting β_2_-AR treatment may imitate some functions and/or effects of exercise.

### Circadian Rhythm

Skeletal muscle, like most other tissues, is known to have a peripheral circadian clock, characterized by the expression of peripheral clock genes. It is thought that the regulation of these peripheral circadian clocks is ultimately regulated and synchronized by a central circadian clock located in the suprachiasmatic nucleus of the brain, however the exact mechanism of communication to skeletal muscle remains unclear.

As previous studies have implicated β-AR signaling as a mediator of circadian rhythm [58-62], it is of interest that we observed the dysregulation of three peripheral clock genes by formoterol administration (Table 1), including nuclear factor, interleukin 3 (*Nfil3*), D site albumin promoter binding protein (*Dbp*), and cryptochrome 2 (*Cry2*). The observation, that only a subset of peripheral clock genes were dysregulated by formoterol administration suggests regulation of these genes is occurring at an organ specific level and not via direct actions on the central circadian clock since interference to the central circadian clock should result in changes to the majority of peripheral clock genes. Furthermore, as only a small fraction of known circadian-regulated genes in skeletal muscle are being altered in our study (compared to McCarthy et al. [63]), this suggests that these results are valid and not occurring due to minor timing issues that occur during tissue collection.

Adrenergic regulation of the related clock gene cryptochrome 1 (*Cry1*) has been previously reported in the rat pineal glands [59,64]. In addition, *Cry1 *has been shown to be regulated by resistance exercise, a stimulus known to increase sympathetic activity in skeletal muscle [65]. This further underscores the crosstalk between exercise, and β_2_-AR signaling in the context of peripheral circadian control.

The central role of circadian rhythm in skeletal muscle metabolism is highlighted by the circadian changes in glucose utilization in muscle, and the findings that disruption of skeletal muscle circadian rhythm occurs in diabetic rats [66]. Furthermore, major changes to peripheral clock genes have been found to be increased by altering the activity of AMP kinase, a key metabolic regulator of skeletal muscle [67]. Interestingly, AMPK modulators have been recently described as exercise mimics [68].

Interestingly, a limited number of genes highlighted in this study have been shown to be regulated in the circadian transcriptome of adult mouse skeletal muscle [63]. *Cry2, Dbp, Ucp3, Pdk4, Ubc, Pgc1α, and Usp2 *have been shown to be regulated in the circadian transcriptome, possibly suggesting that these metabolic transcripts and *Usp2 *may be regulated by dysregulated clock genes rather than directly by β-AR signaling. However, some genes such as *Pgc1α *have a well characterized induction pathway involving β-AR signaling independent of circadian regulation [69].

In conclusion, this is the first paper to show regulation of the peripheral circadian regulators in skeletal muscle by β-AR signaling, possibly implicating β-AR (sympathetic) signaling as a pathway that coordinates communication between central and peripheral circadian clocks in skeletal muscle.

### Transcription and histones

Spurlock et al. [30] have previously used microarray technology to examine skeletal muscle gene expression following chronic β-AR administration. In this study the authors found an up-regulation of transcriptional and translational initiators responsible for increasing protein synthesis. In this context, we observed the induction of 11 genes (Table 1) noted as transcription factors (although some transcription factors are in other categories eg. *FoxO1*). The 11 induced transcriptional regulators were FBJ osteosarcoma oncogene (Fos), kruppel-like factors 2 and 4 (*Klf2 *and *4*), cAMP responsive element modulator (*Crem*), CAAT/enhancer binding protein beta (*Cebpb*), nuclear receptor related 1 protein (*Nurr1*), fos-like antigen 2 (*Fosl2*), V-maf musculoaponeurotic fibrosarcoma oncogene family protein F (*Maff*), activating transcription factor 3 (*Aft3*), T-box 3 (Tbx3), transcript variant 2, and LPS-induced TN factor (Litaf).

Interestingly, concomitant with transcriptional induction, our Illumina analysis revealed that β_2_-AR stimulation significantly inhibited the expression of two histone proteins (also four more histones were present at >2 fold, but these were removed via multiple testing correction), suggesting that β-AR stimulation promote the formation of euchromatin, thus enhancing transcription. This result, coupled with the induction of transcription factors, indicates β-AR signaling may play an important role in regulating skeletal muscle transcription.

### Oxidative stress

In our study, many of the largest inductions occurred in genes associated with the response to oxidative stress. Metallothioneins 1 and 2 (*Mt1*, *2*), sulfiredoxin 1 homolog (*Npn3*), and uncoupling protein 3 (*Ucp3; *listed under metabolism) were all significantly induced at four hours after formoterol administration. During exercise, reactive oxygen species (ROS) are generated in skeletal muscle [70,71], and some oxidative damage occurs [72]. Excess ROS may lead to cellular damage, which has been implicated in with the development of insulin resistance [73], and apoptosis of myoblasts [74]. Given that exercise induces β-AR signaling activity in skeletal muscle [75], this activity may provide a mechanism for increased expression of antioxidant genes to restore oxidative homeostasis. This is supported by the work of Mahoney et al [57], who demonstrated that exercise induced a wide range of antioxidant genes, including several metallothioneins. Furthermore, induction of both *Ucp2 *and *Ucp3 *has also been shown previously to be induced by β-AR signaling in exercising skeletal muscle [27,76-78]. Since *Ucp3 *and *Ucp2 *have been implicated in the reduction of ROS [79,80], it is possible that the induction of *Ucp3 *and *Ucp2 *expression in skeletal muscle is one mechanism for reducing ROS production during or after exercise.

## Conclusion

This study utilized gene expression profiling to examine global gene expression in skeletal muscle following acute and long-term (chronic) β-AR agonist administration. In summary, systemic administration of formoterol had a profound effect on global gene expression in skeletal muscle. With respect to skeletal muscle hypertrophy, formoterol altered the expression of several genes associated with the attenuation of myostatin signaling, highlighting the role of β-AR signaling in the mechanisms regulating skeletal muscle mass

Interestingly, many changes in gene expression with β-AR signaling are similar to findings from other studies examining exercise-related effects in skeletal muscle. For example, the changes in expression of *Pgc1α, FoxO1, Ucp3*, the NR4A subgroup and severalmetallothioneins are identical in both systems [57,65]. This would suggest the induction of β-AR signaling during exercise is a major determinant of the subsequent changes in gene expression changes following exercise.

The findings also demonstrate for the first time crosstalk between β-AR signaling and the peripheral regulators of circadian rhythm in skeletal muscle, possibly implicating β-AR signaling in circadian effects on skeletal muscle. Gene expression changes discovered in the present study may provide insight into the mechanisms underlying β-AR-mediated changes in skeletal muscle hypertrophy and metabolism.

## Methods

### Animals, β-AR agonist administration and tissue collection

All procedures were approved by the Animal Experimentation Ethics Committees of The University of Melbourne. All procedures conformed to the Guidelines for the Care and Use of Experimental Animals described by the National Health and Medical Research Council of Australia. Male C57BL/10 ScSn (wild type, 6-7 weeks old) were obtained from the Animal Resource Centre (Canning Vale, WA, Australia) and were randomly assigned to either saline control, formoterol (a specific β_2_-AR agonist) treated or non-treated groups. For BeadArray analysis, 16 mice in total were analyzed with eight mice for both timepoints. At each timepoint, four mice were treated with formoterol and four with saline (n = 4 mice per timepoint per treatment). For quantitative real-time PCR (qRT-PCR) analysis, ten mice were used per timepoint with five formoterol treated and five saline formoterol animals. Five mice were also analyzed as non-treated animals (n = 5 mice per timepoint per treatment). The animals used for quantitative real-time PCR were independent animals to that used for BeadArray analysis. The mice were housed in the Biological Research Facility at The University of Melbourne and maintained on a 12 h-light/12 h-dark cycle, with standard mouse chow and water provided ad libitum.

Treated mice received a single intraperitoneal injection of formoterol (Astra-Zeneca: 100 μg/kg in 0.2 mL saline) and control mice received an equivalent volume of sterile saline. We have demonstrated previously that this is the most efficacious dose for eliciting skeletal muscle hypertrophy in rodents [3]. For chronic treatment timepoints, mice received a single intraperitoneal injection of formoterol (Astra-Zeneca: 100 μg/kg in 0.2 mL saline) daily and control mice received an equivalent volume of sterile saline daily.

Mice were anesthetized at 1, 4, 8, and 24 hours after acute treatment with formoterol and at 7 and 28 days after chronic formoterol administration. Following anesthetization, tibialis anterior muscles were surgically excised. Tissue was also removed from anesthetized untreated mice (n = 5) that did not receive an intraperitoneal injection for qRT-PCR "no treatment" controls. Tissue from "no treatment" was removed at time zero following injections of the treatment mice. All injections were carried out at approximately 3 hours within the light cycle and muscle was removed at relative appropriate intervals during the light cycle. Care was taken to administer formoterol and saline injections as close as possible to negate gene expression changes associated with circadian rhythm. For chronic treatments injections, were carried out at approximately 3 hours within the light cycle and muscle was removed 24 hours following the last injection. Due to concerns about stress hormones in acute timepoints, both saline control and formoterol treated animals were handled and injected in the same manner. Non-treated animals were also included with the qRT-PCR data to provide an indication of any stress related gene expression changes. As the non-treated animals were quickly anesthetized, stress related gene expression changes should be avoided.

All samples were snap-frozen in liquid nitrogen and stored at -70°C. Samples were used for RNA extraction for BeadArray analysis and qRT-PCR. Contralateral tibialis anterior muscle samples were also used for Western blot analysis.

### RNA extraction for quantitative real-time PCR

RNA was extracted from skeletal muscle tissue using TRI-Reagent (Sigma Aldrich) according to the manufacturer's protocol. For qRT-PCR only, RNA was treated with 2U of Turbo DNase (Ambion) for 30 minutes. RNA was further purified using a mini-RNeasy kit (QIAGEN) according to manufacturer's instructions and quantified using a NanoDrop ND-1000 spectrophotometer.

### cDNA synthesis for quantitative real-time PCR

cDNA was synthesized from 3 μg of total RNA for cell culture experiments and 1 μg for animal muscle experiments (normalized via UV spectroscopy) using Superscript III primed by random hexamers (Geneworks), according to the manufacturer's instructions (Invitrogen).

### Quantitative real-time PCR (qRT-PCR) and statistical analysis

Target cDNA levels were compared by qRT-PCR in 25 μl reactions containing either 1× SYBR green (Applied Biosystems) or Taqman PCR master mix (Roche Molecular Systems), 100 nM of each forward and reverse primers for SYBR green or 1× Assay-on-Demand Taqman primers (Applied Biosystems) and the equivalent of 0.3 μL cDNA. Using an ABI Prism 7500 (Applied Biosystems) sequence detection system, PCR was conducted over 45 cycles of 95°C for 15 seconds and 60°C for 1 minute, preceded by an initial 95°C for 10 minutes. Expression levels were normalized to 36B4 as determined from the ratio of delta CT values. All 36B4 probes remained stable during Illumina BeadArray analysis, confirming this gene was appropriate for normalization. Results are expressed as means ± SEM from five biological replicates. Statistical analyses were performed using GraphPad Prism software. All qRT-PCR data were analyzed using a one-way ANOVA with Bonferroni's post-test.

### qRT-PCR primers

Primers for qRT-PCR analysis of the mRNA populations using SYBR green have been described in detail for 36B4 [81], *Ucp3 *([82], *Nurr1 *[83], *Pdk4 *[28], and *Pgc1α *([82]. The following SYBR primers were designed using Primer Express (Applied Biosystems, Foster City, CA): *Sorbs1 *(F:5'-GTG CCA CAG AAC GAT GAT GAG T-3'; R:5'-AAG TAC CAA ACT GCC TCG TCC TT-3'), Id1 (F:5'-GCA GGT GAA CGT CCT GCT CTA-3'; R:5'-TCT CCA CCT TGC TCA CTT TGC-3'), Activin receptor IIB (F:5'-ACG TGG CGG AGA CGA TGT-3'; R:5'-GTG AGG TCG CTC TTC AGC AG TAC-3'), *Hk2 *(F:5'-TTA GGT CAG TCG GCG TTT CAG-3'; R:5'-TAG GAG GGC AAA TAA ATG TAC AAA CA-3'), *Nfil3 *(F:5'-CGG TTA CAG CCG CCC TTT-3'; R:5'-GTT GTC CGG CAC AGG GTA AAT-3'), and *Stat3 *(F:5'-GAG GAG GCA TTT GGA AAG TAC TGT A-3'; R:5'-GTC ACA CAG ATG AAC TTG GTC TTC A-3'). Assay-on-Demand Taqman primer/probe sets were used to assay expression of myostatin, *Crem*, *Crebp*, *Dbp*, *FoxO1*, and *Lipin1α*.

### BeadArray hybridization and statistical analysis

For BeadArray analysis, 16 mice in total were analyzed with eight mice for both timepoints. At each timepoint, four formoterol treated and four saline formoterol animals. Total skeletal muscle RNA was assessed for integrity using the Agilent Bioanalyzer 2100 and RNA integrity (RIN) scores above 8.3 were present in all samples. 500 ng of RNA was amplified using the Illumina TotalPrep RNA Amplification kit (Ambion) with an in vitro transcription reaction period of 12 hours. Biotinylated, amplified cRNA was assessed for quantity and quality also using the Agilent Bioanalyzer 2100. 1500 ng per array of amplified cRNA was hybridized to Sentrix Mouse-6.v1 BeadChip arrays (Illumina) according to manufacturer directions. Hybridized BeadChip arrays were stained with Amersham fluorolink streptavidin-Cy3 (GE Healthcare). BeadChip arrays were scanned with Illumina BeadStation Scanner and data values with detection scores were compiled using BeadStudio v1.5.1.3 (Illumina) and imported into GeneSpring GX v7.3.1 (Agilent) for data analysis. Mouse Illumina probe set was defined in the GeneSpring Workgroup using the Illumina targetIDs as the unique identifiers and annotated according to array content files supplied by Illumina. Normalized data was produced using GeneSpring GX version 7.3.1 via normalization to control genes, where control genes were represented by all genes with an Illumina detection score equal to one in at least four out of the 16 samples (7,596 control genes in total). All probes except for the 7,596 probes that were determined to have an Illumina detection score equal to one in at least four out of the 16 samples were filtered out to remove probes without adequate expression levels. A parametric Welch's t-test (where variances were not assumed equal) was performed on the 7,596 probes independently for both one and four hour times groups with a p-value cutoff of 0.05. Multiple testing correction (Benjamini and Hochberg False Discovery Rate) was then applied to genes that had passed the parametric Welch's t-test based on the total detected probe-set of 7,596 probes to reduce false positives. About 5.0% of the identified probes would be expected to pass the restriction by chance. Following this statistical filtering, 393 probes were significant at four hours and 43 probes at one hour. Statistical filtered probe-sets were then independently filtered by fold change using a minimum cutoff of 1.85 fold. Following this, 112 probes were present at four hours and 23 genes at one hour. Multiple significant probes for the same gene were removed from final data tables with the probe with the highest fold change being chosen.

### Western blot analysis

Whole skeletal muscle lysates were prepared from homogenisation and sonication in lysis buffer [50 mM Tris HCl, 75 mM NaCl, 5 mM EGTA, 1 mM dithiothreitol, 1% Nonidet P-40, Complete protease inhibitors (Roche Diagnostics Australia), and PhosSTOP phosphatase inhibitors (Roche Diagnostics Australia)] and resolved on 10% SDS-PAGE gels under reducing conditions as outlined [84]. Proteins were transferred to an Immobilon-P polyvinyl difluoride membrane (Millipore) and blocked for 1 h with 5% non-fat milk powder in tris-buffered saline with 0.1% Tween 20. Blots were probed overnight with anti-*Myostatin *(sc-6884; Santa Cruz) at 1:300 and anti-*Gapdh *(R&D Systems) at 1:10000 in blocking buffer. Blots were also probed overnight with anti-*Smad3 *(#9523; Cell Signaling) at 1:1000 and anti-phospho-*Smad3 *(#9520; Cell Signaling) at 1:1000 in 5% fraction V BSA (Sigma-Aldrich) in tris-buffered saline. Secondary anti-rabbit-horseradish peroxidase conjugate (Pierce Biotechnology) in blocking buffer was used at 1:10000 for *Gapdh*, *Smad3*, and phospho-*Smad3 *for 1 h. Secondary anti-goat-horseradish peroxidase conjugate (Pierce Biotechnology) in blocking buffer was used at 1:10000 for *Myostatin *for 1 h. Horseradish peroxidase localization was detected with Immobilon Western Chemiluminescent HRP Substrate (Millipore) according to the manufacturer's instructions and visualized by X-ray film.

## Authors' contributions

MAP was involved in the project conception, designed experiments, carried out RNA extraction, performed qRT-PCR and Western blot analysis, performed data analysis, analyzed Illumina data, and drafted the manuscript. JGR carried out animal handling, formoterol treatments and animal dissection and contributed to preparing the manuscript. JGR and GSL contributed to the study design and in the preparation of the manuscript. GEOM was involved in the project conception, experimental design, data analysis and manuscript preparation. All authors read and approved the final manuscript.
